# Primary central nervous system small lymphocytic lymphoma in the bilateral ventricles: two case reports

**DOI:** 10.1186/s12883-019-1430-3

**Published:** 2019-08-19

**Authors:** Rongjing Guo, Xiaolong Zhang, Chunxiao Niu, Yibin Xi, Hong Yin, Hong Lin, Ting Chang

**Affiliations:** 1 Department of Neurology, Tangdu Hospital, the Air Force Medical University, 569 Xinsi Road, Xi’an, Shaanxi Province 710038 People’s Republic of China; 2 Department of Radiology, Xijing Hospital, the Air Force Medical University, Xi’an, Shaanxi Province People’s Republic of China

**Keywords:** Small lymphocytic lymphoma, Non-Hodgkin lymphoma, Central nervous system, Bilateral ventricular neoplasm

## Abstract

**Background:**

Primary central nervous system (CNS) small lymphocytic lymphoma (SLL), as a type of low-grade lymphoma, is extremely rare. The diagnosis of CNS SLL is challenging due to its variable clinical and radiological features, which may overlap with those of diffuse large B-cell lymphoma (DLBCL). Primary CNS SLL differs from DLBCL in that it has an indolent clinical course and a good prognosis. Thus, it is important to distinguish SLL from DLBCL. By reviewing the literature, only two cases of low-grade SLL, primarily located in the CNS and involving the brain parenchyma and dura, have been reported. To our knowledge, primary CNS SLL in the bilateral ventricles has never been reported. Interestingly, the two cases in our report are identical in terms of the clinical presentations, magnetic resonance imaging (MRI) features, pathological results and prognoses.

**Case presentation:**

Both patients presented with headaches. MRI suggested solid lesions located in the bilateral ventricles that were isointense on T1-weighted images and hypointense on T2-weighted images. After the injection of contrast agent (gadolinium, Gd), the intraventricular lesions were homogeneously enhanced and hyperperfused. CSF cytology revealed malignant cells. Brain biopsy revealed diffuse proliferation of small lymphocytes with positive labelling of B-cell immunomarkers. The primary origin in the CNS was confirmed with no evidence of systemic lymphoma. Two patients were given high doses of methotrexate-based chemotherapy and were free from symptoms and progression for more than 1-year of follow-up.

**Conclusions:**

The presence of homogeneously enhanced intraventricular MRI lesions should raise the suspicion of primary CNS SLL.

## Background

Primary central nervous system lymphoma (PCNSL) is a rare type of extra-nodal non-Hodgkin lymphoma (NHL) that can be found in the brain, leptomeninges, eyes or spinal cord without evidence of systemic lymphoma. PCNSL constitutes 4% of primary brain tumours, and approximately 90% of cases are aggressive, diffuse large B-cell lymphoma (DLBCL) with a poor outcome. Other lymphoma categories, such as low-grade subtypes and T cell lymphomas, are extremely rare [1].

Small lymphocytic lymphoma (SLL), as a type of low-grade NHL, is different from DLBCL in that it has an indolent clinical course, has less aggressive features and has a good prognosis [2–4]. Therefore, it is important to distinguish SLL from DLBCL. However, the diagnosis of primary SLL is challenging due to its variable clinical and radiological features, which may overlap with DLBCL and other primary CNS malignant tumours. Here, for the first time, we report two cases of primary CNS SLL located in the bilateral ventricles with identical clinical presentations, MRI features, pathological results and prognoses.

## Case presentation

### Patient 1

**Fig. 1:**
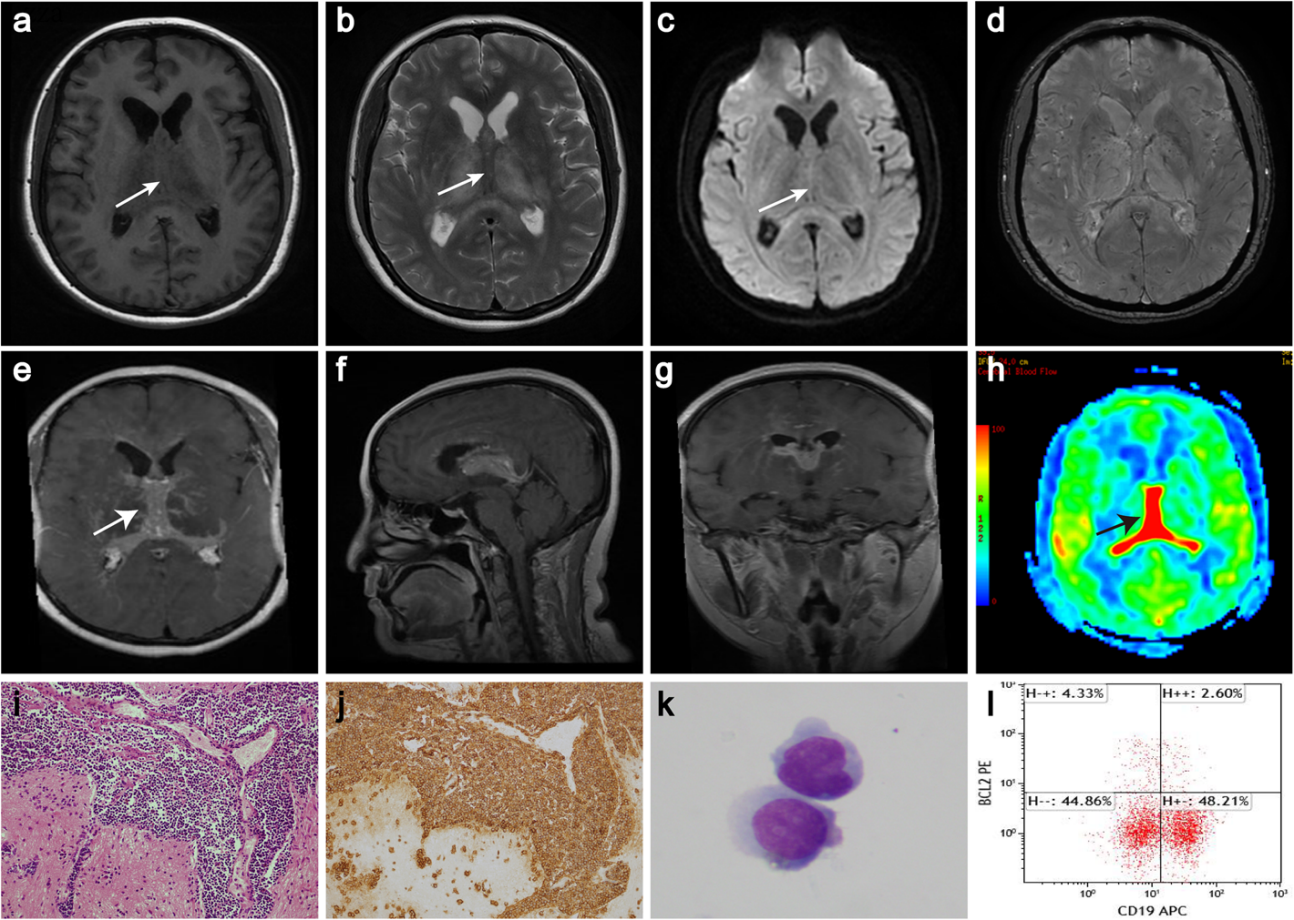
The solid lesions are located in the bilateral ventricles and fornix, presenting as isointense on T1WI (**a**, white arrow) and hypointense on T2WI (**b**, white arrow) without restricted diffusion (**c**, white arrow on DWI). Axial SWI demonstrates enlarged veins in the thalamus and located adjacent to the lateral ventricles (**d**). The lesions demonstrate homogeneous contrast enhancement (**e**-**g**, axial (white arrow), sagittal, and coronal T1 with contrast) and hyperperfusion on 3D-ASL MR perfusion imaging (**h**, black arrow). Open biopsy of the lesions shows diffuse proliferating small lymphocytes (H&E × 200) (**i**). CD-20 positivity of lymphocytes (× 200) (**j**). CSF cytology reveals malignant cells, and flow cytometry immunophenotyping reveals a lymphocyte subset with the profile of CD19^+^ (50.81%) (**k** and **l**)

**Fig. 2:**
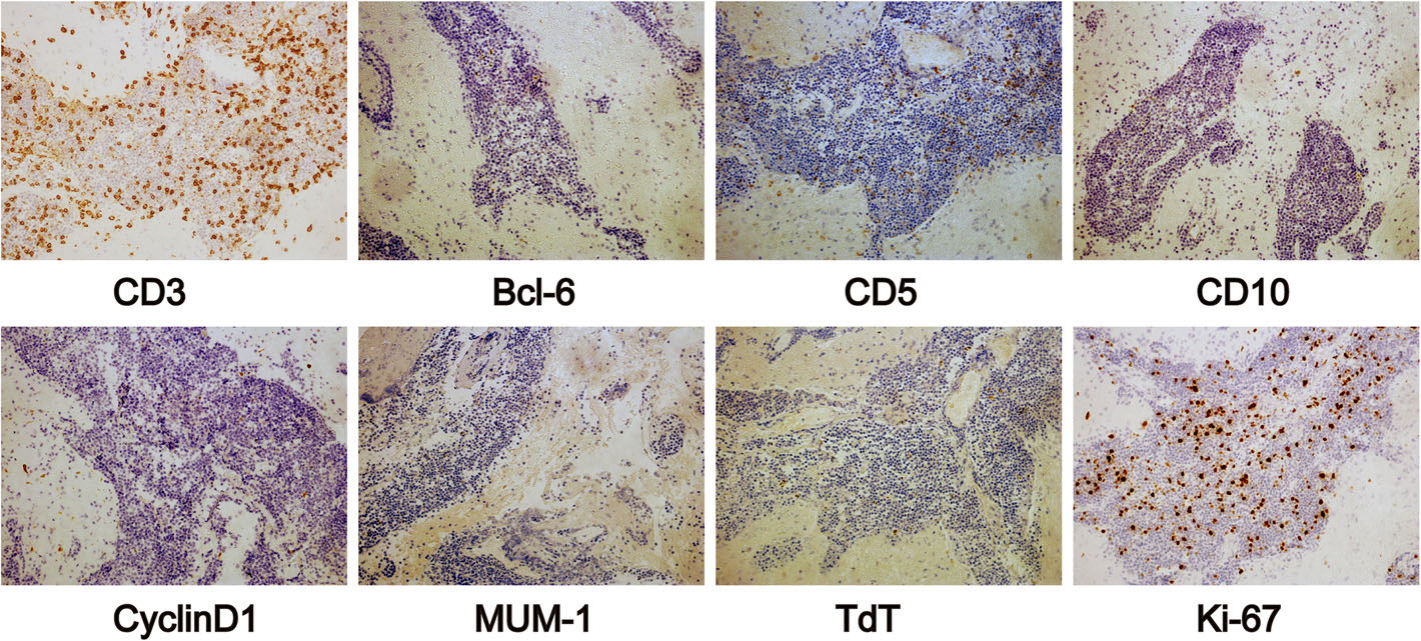
Lymphocytes are partially positive for CD3 and non-reactive to bcl-6, CD5, CD10, CyclinD1, multiple myeloma oncogene 1 (MUM-1) and TdT. Ki-67-positive lymphocytes suggest of low proliferation index (less than 7%). (× 200)

A 45-year-old woman presented to the neurological department with a 6-month history of headache. The patient described her headache as generalized, slightly dull, not persistent and without any exacerbating or relieving factors. She denied fever, dizziness, nausea or vomiting. She also had no ocular complaints such as blurred vision, floaters, decreased acuity, pain, photophobia and diplopia. There was also no history of cognitive decline and personality changes, seizures, loss of coordination and gait disorders, immunosuppressive drug intake, radiation exposure, and systemic infection or other autoimmune diseases. Her family history was unremarkable. Neurologic examination did not show any focal neurological signs. MRI demonstrated solid lesions in the bilateral ventricles and fornix, which were isointense on T1-weighted images (T1WI) and hypointense on T2-weighted images (T2WI) without diffusion restriction in diffusion-weighted imaging (DWI). Furthermore, the thalamus and callosal splenium demonstrated bilaterally symmetric swelling and hyperintensity on T2WI (Fig. 1a-c). Enlarged veins in the bilateral thalami and located adjacent to the surfaces of the bilateral ventricles were observed on axial susceptibility-weighted imaging (SWI) (Fig. 1d). The lesions demonstrated homogeneous enhancement on postcontrast T1WI (Fig. 1e-g) and hyperperfusion on 3D-arterial spin-labelling (ASL) MR perfusion images (Fig. 1h). Brain transcallosal biopsy was performed. The lesions were found to be located in the roof of the third ventricle, and they extended along the fornix from rostral to caudal and enveloped the cerebral internal veins, infiltrating the bilateral thalami and the choroid fissure of the lateral ventricles. Haematoxylin-eosin (H&E) staining showed diffuse proliferation of small lymphocytes in the biopsy tissue (Fig. 1i). Immunostaining demonstrated positive labelling for the B-cell immunomarker CD20 (Fig. 1j). In addition, CD20 and other B-cell immunomarkers such as CD79a and Pax-5 were found to be positive, and lymphocytes were shown to be partly positive for the T-cell immunomarker CD3. Bcl-6, CD2, CD5, CD10, CD7, CD23, CD34, NF, TdT, multiple myeloma oncogene 1 (MUM-1) and glial fibrillary acidic protein (GFAP) were found to be negative in tumour cells. The mantle cell lymphoma marker CyclinD1 was also non-reactive. The proliferation index was assessed by applying Ki-67 antibody and was found to be low (less than 7%). Partial immunomarkers are shown in Fig. 2. Rearrangement of the immunoglobulin gene was revealed. A lumbar puncture was performed, and cerebrospinal fluid (CSF) showed 42 white cells (normal range, < 4*10^6^/L) and significantly elevated CSF protein at 1773.1 mg/L (normal range, 80–430 mg/L) and IgG at 235 mg/L (normal range, 0–34 mg/L). CSF cytology revealed malignant cells (Fig. 1k), and flow cytometry immunophenotyping analysis demonstrated a lymphocyte subset with the profile of CD19^+^ (50.81%) (Fig. 1l). Testing for syphilis, HCV and HIV was negative. Further, bone marrow aspiration cytology and histology examinations as well as thoracic and abdominal CT and ophthalmology examinations with slit-lamp were unremarkable, and clinical examination revealed no lymphadenopathy or organomegaly, confirming the ‘primary’ origin of CNS lymphoma. The diagnosis of SLL in the bilateral ventricles was confirmed based on the pathological results. The patient was treated with methotrexate (7 g) and dexamethasone chemotherapy in a total of 5 cycles from Feb to July 2018 without radiotherapy. Simultaneously, intrathecal methotrexate (15 mg) and cytarabine (50 mg) were also administered. Her headache completely resolved after the last chemotherapy session. Simultaneously, follow-up CSF studies showed 4 white cells (reference range: < 4*10^6^/L) and slightly elevated CSF protein (623.4 mg/L; reference range: 80–430 mg/L). Simultaneously, follow-up MRI showed that the lesions were similar in terms of their location, size and shape compared with prechemotherapy values. Thereafter, she was followed as an outpatient and underwent MRI every 3 months. The last follow-up was performed in May 2019. The patient described no clinical symptoms, and there was no significant change in the lesions compared with prechemotherapy values. The overall follow-up period was more than 1 year.

### Patient 2

**Fig. 3:**
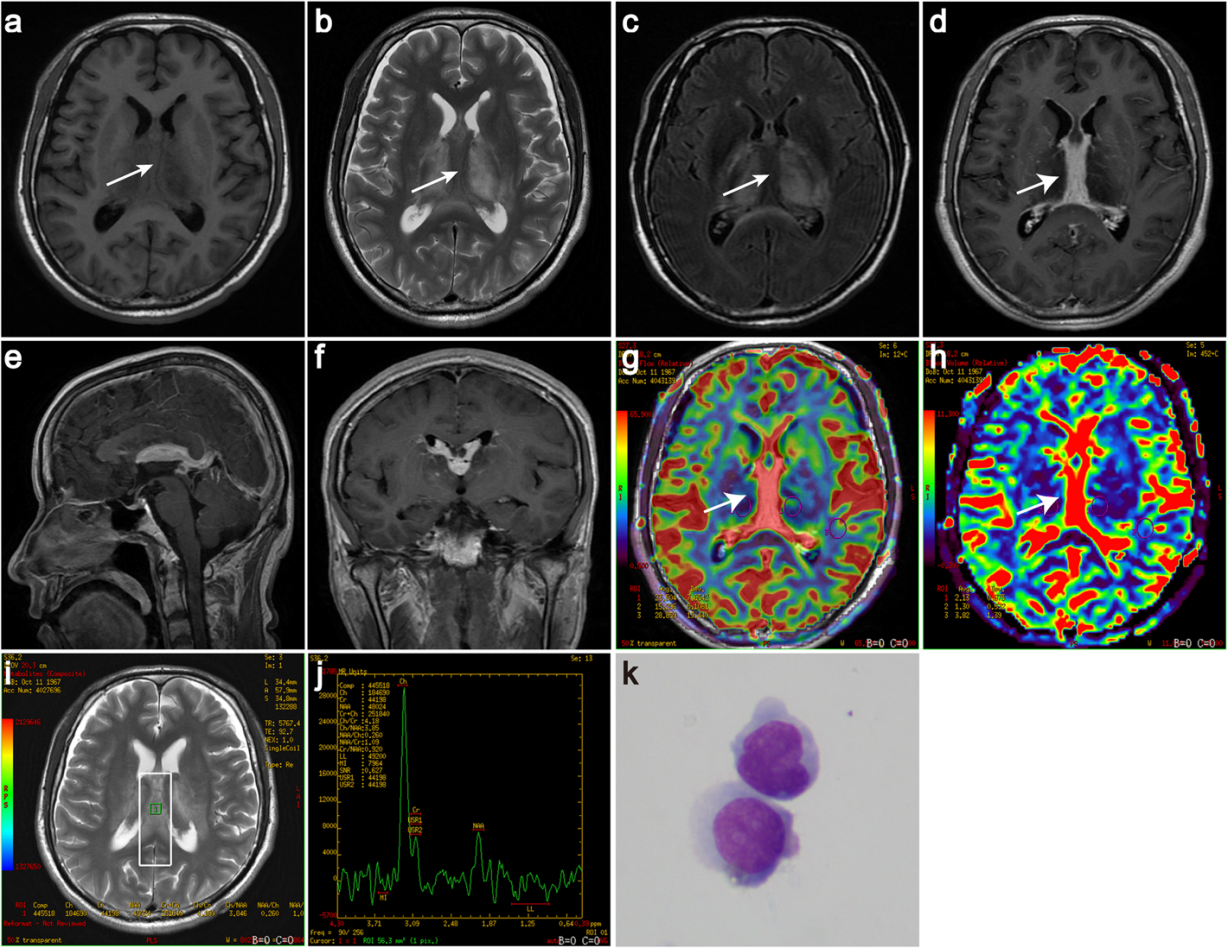
The solid lesions in the bilateral ventricles presenting as isointense on T1WI (**a**, white arrow) and hypointense on T2WI and FLAIR images (**b** and **c**, white arrow). Striking contrast enhancement (**d**-**f**, axial (white arrow), sagittal, and coronal T1 with contrast) and hyperperfusion are demonstrated in the lesions of the bilateral ventricles (**g** and **h**, white arrow, rCBF, rCBV map). MRS from the lesion demonstrates a high Cho peak and a low NAA peak with a Cho/NAA ratio of 3.85 (**i** and **j**). CSF cytology reveals malignant cells (**k**)

A 49-year-old man presented to the neurological department with headache and dizziness for the past 5 years. He described an episodic left-sided headache without aura. Each symptom lasted 2 min, and there had been 2–3 attacks within 1 month. The symptoms were not severe, and there was no interference with daily routines. He denied fever, nausea, vomiting, or neck stiffness. He also had no visual symptoms and no history of cognitive decline, personality changes, and focal neurological deficits. His family history was unremarkable. Neurologic examination did not show any focal neurological signs. MRI revealed solid lesions involving the bilateral ventricles, thalamus and callosal splenium. Intraventricular lesions were isointense on T1WI (Fig. 3a). On T2WI and fluid-attenuated inversion recovery (FLAIR) images, hyperintense abnormalities were identified at the site of the lesions (Fig. 3b-c). After the injection of contrast agent, the intraventricular lesions were homogeneously enhanced (Fig. 3d-f, axial, sagittal, coronal T1 with contrast), and hyperperfusion was shown on the MR perfusion images in Fig. 3g and h for rCBF (relative cerebral blood flow) and rCBV (relative cerebral blood volume), respectively. The MR spectra from the intraventricular lesions demonstrated a significant increase in the choline (Cho) peak and a reduction in the N-acetyl aspartate (NAA) peak with a Cho/NAA ratio of 3.85 (Fig. 3i and j). CSF studies showed 36 white cells (reference range: < 4*10^6^/L) with 82% lymphocytic cells and significantly elevated protein (4424.24 mg/L; reference range: 80–430 mg/L) and IgG at 286 mg/L (reference range: 0–34 mg/L). CSF cytology revealed malignant cells (Fig. 3k). A stereotactic biopsy of the brain showed a dense cellular infiltration of small round lymphoid cells co-expressing CD20 and Pax-5 but not CD3, CD10, bcl-6, MUM-1 and c-myc. The Ki-67 proliferation marker was up to 10%. The patient’s HIV screening test was negative. Further, bone marrow aspiration cytology and histology examinations as well as thoracic and abdominal CT and ophthalmology examinations with slit-lamp showed no evidence of systemic lymphoma. He completed 4 cycles of high-dose methotrexate (8 g)-based chemotherapy (rituximab, methotrexate, dexamethasone and vincristine) without radiotherapy. Intrathecal methotrexate was also administered. The patient’s symptoms were evaluated through telephone follow-up. The last follow-up was performed in May 2019. He was free from symptoms and progression, but a follow-up MRI was not available.

## Discussion and conclusions

Primary CNS SLL is extremely rare. By review of the literature, only two cases of primary SLL involving the brain parenchyma and dura have been reported so far [5, 6]. In this study, for the first time, we reported two cases of primary SLL located in the bilateral ventricles with identical clinical presentations, neuroimaging features, pathological results and prognoses.

The main presenting symptom of these two cases was a slight headache without cognitive impairment, abnormal behaviour, personality changes or ocular complaints that are frequently observed in DLBCL. In addition, these 2 patients were free from symptoms and progression after high-dose methotrexate-based chemotherapy for over one-year of follow-up. These results indicate that the clinical course of primary CNS SLL is indolent and less aggressive, which is consistent with a report from Jahnke and colleagues [4]. Among their retrospective cohort, 20 patients with low-grade small lymphocytic lymphoma were included. Their long-term results revealed a relatively indolent clinical course and a long survival of patients with low-grade PCNSL compared with patients with high-grade PCNSL. The prognosis results in our patients require longer-term observations. High-grade PCNSL usually demonstrates very high proliferative activity with a Ki-67 index of 70–90%. However, low-grade PCNSL demonstrates a low proliferation index, suggesting its less-aggressive course compared with high-grade PCNSL.

Due to the rarity of low-grade PCNSL and the limited data derived from a few case reports and pathological series, no standard therapy is currently available. Most treatment protocols come from the results of high-grade PCNSL. Front-line therapy for high-grade PCNSL consists of high-dose methotrexate-based poly-chemotherapy. In addition, radiotherapy alone or in combination with chemotherapy is always considered [7]. Our patients received high-dose methotrexate-based chemotherapy. Jahnke’s study proposed that less-aggressive treatment might be adopted for low-grade PCNSL due to its relatively indolent clinical course [4].

Radiologically, in contrast to DLBCL and other reports of SLL, the lesions in the present cases showed hypointense T2WI, isointense T1WI and homogeneous enhancement [8, 9]. As is well known, cases of low-grade PCNSL frequently demonstrate absent/moderate and irregular post-contrast enhancement compared to cases of high-grade PCNSL. It is interesting that these two cases demonstrated homogeneous enhancement. Thus, we speculated that such neuroimaging features might be typical of primary SLL in the bilateral ventricles. In addition, the bilateral thalami and callosal splenium showed symmetric swelling and hyperintensity on T2WI in the absence of contrast enhancement. We presumed that intraventricular lesions obstructed the drainage of the vein of Galen and consequently contributed to bilateral thalamic swelling and hyperintensity. The vein of Galen, consisting of the internal cerebral veins and basal veins, drains the deep white matter, the corpus callosum, and the basal ganglia. We further observed enlarged veins in the thalamus on SWI. Bilateral thalamic swelling makes it easy to confuse the diagnosis and to ignore intraventricular lesions.

Low-grade PCNSL with homogeneously enhancing intraventricular lesions should be differentiated from other common ventricular neoplasms, such as choroid plexus papilloma, meningioma, subependymoma and metastasis. In addition, differential diagnosis must be made with other types of PCNS lymphoma involved in the ventricles [10, 11]. Although advanced imaging techniques can increase the diagnostic accuracy and help in differentiating PCNSL from other tumours or non-tumour lesions, brain biopsy is required to confirm the diagnosis.

In conclusion, the presence of homogeneously enhanced intraventricular MRI lesions should raise the suspicion of primary CNS SLL.

## Data Availability

Data sharing is not applicable to this article because no datasets were generated or analysed during the current study.

## Abbreviations

PCNSL: Primary central nervous system lymphoma
NHL: Non-Hodgkin lymphoma
DLBCL: Diffuse large B-cell lymphoma
SLL: Small lymphocytic lymphoma
MRI: Magnetic resonance imaging
T1WI: T1-weighted imaging
T2WI: T2-weighted imaging
CSF: Cerebrospinal fluid
MUM-1: Multiple myeloma oncogene 1
GFAP: Glial fibrillary acidic protein
ASL: Arterial spin labelling
SWI: Susceptibility-weighted imaging
FLAIR: Fluid attenuated inversion recovery
